# Beyond the pitch: The unaddressed challenges of inadequate uniforms in women's football

**DOI:** 10.17179/excli2025-9263

**Published:** 2026-02-10

**Authors:** Rizia Rocha-Silva, Geovana José, Marília Santos Andrade, Claudio Andre Barbosa de Lira

**Affiliations:** 1 Center for Teaching and Research Applied to Education, Federal University of Goiás, Goiânia, Brazil; 2 Faculty of Information and Communication, Federal University of Goiás, Goiânia, Brazil; 3 Department of Physiology, Federal University of São Paulo, São Paulo, Brazil; 4 Faculty of Physical Education and Dance, Federal University of Goiás, Goiânia, Brazil

## ⁯

In recent decades, we have witnessed the welcome increase in women's participation in both recreational and professional sport. Public policies and specific legislation have played a crucial role in this progress. For example, Title IX of the Education Amendments of 1972 (USA), which prohibits gender discrimination in government-funded educational institutions, led to a substantial rise in female participation in school and university sports (U.S. Department of Justice, 1972[9]). The most recent milestone in women's participation was reached at the Paris 2024 Olympic Games, the first edition in which the number of female athletes equaled that of male athletes (International Olympic Committee, 2024[2]). Yet, despite these advances, certain aspects of sport still fail to consider the specificities of the female body. One striking example is that female athletes continue to be subjected to uniforms conceived under a logic that disregards their anatomical and physiological needs.

It is widely recognized that sex-related physiological differences exist. On average, women present lower maximal oxygen uptake, lower muscle mass and strength, distinct body morphology, and different weight distribution (Landen et al., 2023[4]). Moreover, the female pelvis is wider, with the acetabula set farther apart, which increases hip width. Paradoxically, the same science that acknowledges such differences is often ignored in the development of uniforms. Frequently, female players are offered nothing more than reduced versions of male models, as if unisex design were synonymous with equality (Okholm Kryger et al., 2022[7]).

The consequences go beyond aesthetics. Reports published in media coverage, such as in Folha de S. Paulo (Macedo, 2025[6]) and growing scientific evidence indicate that inadequate uniforms compromise performance, increase the risk of injury, and generate anxiety related to menstrual leakage (Okholm Kryger et al., 2022[7]; Krumer, 2024[3]). In elite football, players wore male uniforms for decades, and only in 2019, during the FIFA Women's World Cup, did national teams receive tailored kits. Yet problems remain: overly short shorts, excessively tight shirts, sports bras that cause breast pain and discomfort (Lofthouse, 2024[5]; Wakefield-Scurr et al., 2025[10]), uncomfortable socks, and boots not adapted to female anatomy, which may contribute to injury risk (Okholm Kryger et al., 2023[8]).

Despite modest advances, such as the abandonment of white shorts and minor adjustments in tailoring, it is evident that definitive solutions will require more than superficial adaptations. While male sportswear has benefited from decades of scientific research aimed at optimizing performance, safety, and comfort, the female counterpart still suffers from a striking lack of investigation. This gap is evident, for example, in the scarcity of systematic studies on thermoregulation or the effects of fabric and garment design on the female athlete's body (Hutchins et al., 2021[1]).

Addressing these shortcomings requires the adoption of evidence-informed strategies. Three priority axes for intervention are proposed. First, co-design approaches should actively involve female athletes in research and development processes, incorporating three-dimensional body scanning and movement analyses that account for sex-specific biomechanical characteristics. Second, regulatory bodies, such as FIFA and International Olympic Committee, should establish minimum ergonomic certification standards to ensure that equipment accommodates morphological diversity, particularly in footwear design and stud configuration, thereby mitigating risk factors associated with anterior cruciate ligament injuries (Okholm Kryger et al., 2023[8]). Third, funding agencies should prioritize interdisciplinary research integrating textile technology, thermoregulation, and biomechanics to strengthen the empirical basis for innovation in women's sport equipment.

To close this debate without acknowledging the historical inequality in sportswear research and development would be to perpetuate neglect. It is time to place the performance and well-being of female athletes at the center of priorities, ensuring equitable conditions between women's and men's sport. The numerical equality achieved in Paris must not conceal the material inequality that continues to dress women's bodies in sport.

## Declaration

### Conflict of interest

The authors declare that they have no competing interests.

### Acknowledgments

The authors declare that no funds, grants, or other support were received during the preparation of this letter.

### Artificial Intelligence (AI) - assisted technology:

Authors used AI-assisted technologies to check for grammatical edits.

### CRediT authorship contribution statement

Conceptualization: RR-S, CABL, Writing - Original Draft: RR-S, GJ, Writing - Review & Editing: MSA, CABL
